# Risk of Stroke in Patients with Breast Cancer and Sleep Disorders

**DOI:** 10.7150/jca.63184

**Published:** 2021-09-21

**Authors:** Nan-Cheng Chen, Kuang-Ming Liao, Yu-Feng Tian, Yu-Cih Wu, Jhi-Joung Wang, Chung-Han Ho, Chien-Chin Hsu

**Affiliations:** 1 Department of Internal Medicine, Chi Mei Medical Center, Chiali, Tainan, Taiwan.; 2 Department of Biotechnology and Food Technology, Southern Taiwan University of Science and Technology, Tainan, Taiwan.; 3 Division of Gastroenterology & General Surgery, Chi Mei Medical Center, Tainan, Taiwan.; 4 Department of Health and Nutrition, Chia Nan University of Pharmacy and Science, Tainan, Taiwan.; 5 Department of Medical Research, Chi Mei Medical Center, Tainan, Taiwan.; 6 Department of Anesthesiology, Chi Mei Medical Center, Tainan, Taiwan.; 7 Department of Anesthesiology, National Defense Medical Center, Taipei, Taiwan.; 8 Department of Information Management, Southern Taiwan University of Science and Technology, Tainan, Taiwan.; 9 Cancer Center, Wan Fang Hospital, Taipei Medical University, Taipei, Taiwan.; 10 Department of Emergency Medicine, Chi-Mei Medical Center, Tainan, Taiwan.

## Abstract

Breast cancer and stroke were leading cause of cancer-related mortality in the world. Stroke is the second leading cause of death. Previous studies showed that patients with breast cancer had a relatively higher risk of sleep disorders. Sleep disorders increased the risk of stroke. The aim of our study was to examine the risk of stroke after a breast cancer with sleep disorder among women in Taiwan.

The Taiwan Cancer Registry was used to identify patients with breast cancer. Patients with new-onset breast cancer from January 2007 to December 2015 were selected for this study and followed until December 31, 2017. Patients who were diagnosed with sleep disorders were set as the case group, and the controls were those without sleep disorders.

We enrolled 5256 patients with sleep disorders and 10,512 patients without sleep disorders. There were 121 (2.30%) patients with ischemic stroke among the breast cancer patients with sleep disorders. The mean time from the diagnosis of breast cancer to the occurrence of ischemic stroke was 6.29±2.59 years for breast cancer patients with sleep disorders and 6.00±2.76 years for those without sleep disorders (p < 0.0001). After matching by age and index year, breast patients with sleep disorders had a 1.31-fold higher risk (95% confidence interval: 1.03-1.66; p-value=0.026) of ischemic stroke than those without sleep disorders, after adjustment for comorbidities, cancer clinical stage, and treatment types. In conclusion, Breast cancer patients with sleep disorders have an increased risk of stroke.

## Introduction

Breast cancer is one the most common cancers diagnosed in women in the United States. There were approximately 2.1 million cases of newly diagnosed breast cancer in women in 2018, accounting for one-fourth of all cancer cases among women 1; breast cancer is also the leading cause of cancer-related mortality in over 100 countries 2. Stroke is the second leading cause of death and is responsible for approximately 11% of all deaths worldwide 3.

Cancer is a risk factor for stroke because of hypercoagulability, direct tumor compression of blood vessels, radiation therapy and chemotherapy 4. Patients with prostate, breast, and colorectal cancer had a higher risk of dying from fatal stroke than patients with other cancers 5. Patients who survived breast cancer had a higher risk of death from stroke than patients who had never had breast cancer. In addition, patients with breast cancer may also have concomitant sleep disorders. A study showed that patients newly diagnosed with breast cancer had a relatively higher risk of sleep disorders 6. Sleep disorders affect patients with cancer. Sleep disorders can result in mood disorders, weakness, and immune dysfunction and can contribute to the risk of stroke 7. Compared to breast cancer, sleep disorders have received scant attention from physicians. In addition to predicting the prognosis of the cancer, it is important to identify cancer survivors at elevated risk of stroke and to determine the impact of sleep disorders on stroke. The aim of our study was to examine the risk of stroke after a breast cancer with sleep disorder among women in Taiwan.

## Material and methods

### Data source

The Taiwan Cancer Registry (TCR) was used to identify patients with breast cancer. TCR was established to register all newly diagnosed cancer patients in 1979. The TCR has information about individual patient demographics, cancer stages, primary cancer sites, tumor histologies, and treatment types. During the different periods, TCR presented the short-form format and long-form format in different major cancers. The related information had been published in the previous research 8-10.

The disease history and related diagnoses among those patients were obtained from the National Health Insurance Research database (NHIRD). NHIRD was a claims database based on Taiwan's single-payer national health insurance program which covered almost 99% population of Taiwan. The information about inpatient and outpatients records, including diseases, procedures, and prescriptions, were all included in NHIRD 11,12. The diagnosis codes were based on the International Classification of Diseases, Ninth Revision, Clinical Modification (ICD-9-CM). For research purposes, the Health and Welfare Data Science Center (HWDC) released the above database to the public in a deidentified and therefore anonymized format. This study was conducted in compliance with the Declaration of Helsinki and approved by the Ethics Committee of the Institutional Review Board of Chi-Mei Hospital (IRB:10912-E02).

### Study population

**Figure 1:**
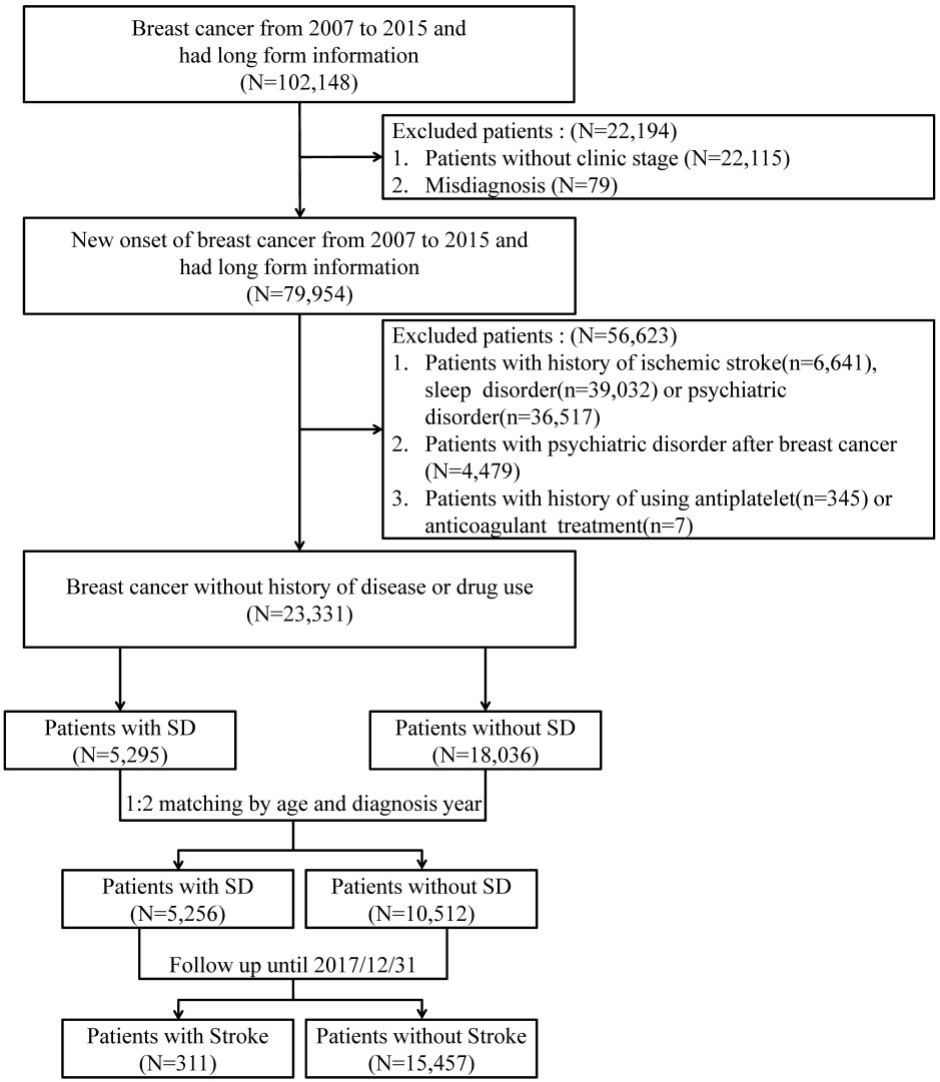
Flowchart of subjects' selection.

Patients with new-onset breast cancer from January 2007 to December 2015 were selected for this study. All patients were followed until December 31, 2017. Patients who were diagnosed with sleep disorders (ICD-9-CM: 327, 307.4, 780.5) were set as the case group, and the controls were those without sleep disorders. According to the study aim of estimating the ischemic stroke risk among patients with new-onset sleep disorders, patients with a history of ischemic stroke (ICD-9-CM: 433-438) before the diagnosis of a sleep disorder, a history of sleep disorders before the diagnosis of breast cancer, or a history of psychiatric disorders (ICD-9-CM: 290-306) were excluded. Considering the potential effects of certain drugs on the outcome of interest, patients with a history of using antiplatelets or anticoagulants were also excluded. In addition, 1:2 age- and index-year matching was used to reduce the baseline difference between cases and controls. Figure 1 presents the flowchart of study subject selection.

### Measurements

The major outcome of this study was ischemic stroke, which was identified as patients diagnosed with ischemic stroke after being diagnosed with breast cancer and sleep disorders. We focus on ischemic stroke because previous studies showed that sleep disorder is a risk factor for acute ischemic stroke 13-15. The following information was collected for the patients with breast cancer: age at diagnosis with breast cancer, clinical stage of cancer, and cancer treatment types. Furthermore, the investigated comorbidities before the diagnosis of breast cancer were hypertension (HTN, ICD-9-CM: 401-405), diabetes mellitus (DM, ICD-9-CM: 250), hyperlipidemia (ICD-9-CM: 270), and coronary artery disease (CAD, ICD-9-CM: 410-429).

### Statistical analysis

The differences in continuous variables and categorical variables between the breast cancer patients with sleep disorders and those without sleep disorders were evaluated using Student's t-test and Pearson's chi-square test, respectively. Logistic regression analysis was used to estimate the association of the risk of stroke with sleep disorders. As matched pairs were investigated in this study, the association between sleep disorders and stroke after a diagnosis of breast cancer was estimated using a conditional logistic regression model. The odds ratios (ORs) with 95% confidence intervals (CIs) were calculated after adjusting for the selected variables. A stratified analysis of selected subjects was also performed using a forest plot. All analyses were conducted using SAS statistical software version 9.4 (SAS Institute, Inc., Cary, NC, USA). The statistical significance was set at a p-value < 0.05.

## Results

In this study, 15,768 patients with breast cancer were enrolled, including 5256 patients with sleep disorders and 10,512 patients without sleep disorders. The baseline information in breast cancer patients with and without sleep disorders is presented in Table 1. The incidence of the outcome of interest, ischemic stroke, was significantly different between breast cancer patients with and without sleep disorders. There were 121 (2.30%) patients with ischemic stroke among the breast cancer patients with sleep disorders and 190 (1.81%) among the breast cancer patients without sleep disorders (P=0.0352). The mean time to the diagnosis of sleep disorders among patients with breast cancer was 2.00±2.10 years. In addition, the mean time from the diagnosis of breast cancer to the occurrence of ischemic stroke was 6.29±2.59 years for breast cancer patients with sleep disorders and 6.00±2.76 years for those without sleep disorders (p < 0.0001).

Table 2 shows the association of the risk of ischemic stroke with sleep disorders. Before matching, breast cancer patients with sleep disorders had a higher risk of developing ischemic stroke than those without sleep disorders (OR: 1.58; 95% CIs: 1.27-1.96; p-value < 0.0001). After matching by age and index year, breast patients with sleep disorders had a 1.31-fold higher risk (95% CIs: 1.03-1.66; p-value=0.0260) of ischemic stroke than those without sleep disorders, after adjustment for comorbidities, cancer clinical stage, and treatment types.

The stratified analysis among selected groups was illustrated in a forest plot after matching. Among breast cancer patients who underwent surgery or chemotherapy, those with sleep disorders had a significantly higher risk of ischemic stroke than those without sleep disorders (OR: 1.36, 95% CI: 1.06-1.73, for surgery; OR: 1.41, 95% CI: 1.04-1.92, for chemotherapy).

## Discussion

To our knowledge, this is the first study to delineate the association between ischemic stroke and sleep disorders in patients with breast cancer. We found that patients with breast cancer and sleep disorders had an elevated risk of ischemic stroke.

### Breast cancer and stroke

Stroke is not rare in patients with cancer and significantly worsens patient condition and prognosis. A previous study found that stroke can occur in cancer patients via a variety of mechanisms, including tumor invasion, hypercoagulability, disseminated intravascular coagulation, the side effects of chemotherapy and radiation therapy, infections, tumor embolism and nonbacterial thrombotic endocarditis 4. Approximately 15% of patients with cancer have cerebrovascular disease 16.

Studies have shown that stroke occurs more frequently in cancer patients than in the general population and can present as the first sign of cancer, leading to its detection 17. Wei et al. 18 found that the risk of stroke was highest between 0.5 years before and after a diagnosis of cancer, with a steep-sided bell curve.

Our study focused on breast cancer and stroke and found that the time from the breast cancer diagnosis to the occurrence of stroke was approximately 6 years. According to Wei et al. 18, the highest risk of stroke was found in patients with malignant brain tumors, followed by those with gastric cancer, prostate cancer, urogenital cancer, lung cancer, and leukemia. These cancer types conferred a higher risk of stroke than breast cancer, and their time from the diagnosis of cancer to the occurrence stroke was shorter.

Jang et al. 19 used the Korean National Health Insurance Service National Sample Cohort database between 2002 and 2015 and followed-up patients for 7 years to survey the effect of cancer on stroke. The results showed that the risk of stroke increased at 3 years after cancer, and the effect was sustained for 7 years. In their study, they divided cancer types according to body systems. These systems included the digestive system, respiratory/intrathoracic system, and “others”. They did not further analyze the associations between specific cancer types and stroke.

### Sleep disorder and stroke

The relationship between sleep disorders and stroke has been well studied. Sleep disorders increase the risks of stroke, all-cause mortality and cardiovascular death 20. There are some mechanisms by which sleep disorders can lead to stroke. Previous studies have shown that most types of sleep disorders, including insomnia, sleep apnea, narcolepsy and periodic limb movements, lead to sleep fragmentation, which is characterized by electroencephalographic arousals or an increase in the level of chin electromyography (in addition to the appearance of an α-rhythm on electroencephalography) in all sleep stages 21.

Sleep fragmentation significantly alters sympathetic activity, and this effect seems to be associated with the time spent in rapid eye movement sleep. Sleep fragmentation has been found to increase postsleep diastolic blood pressure and dysregulation of the autonomic nervous system, further leading to endothelial dysfunction and increased arterial stiffness as cardiovascular consequences 22.

Sleep fragmentation increases the sleep/wake blood pressure ratio, increases the proportion of time spent in light sleep and decreases the proportion in time spent in rapid eye movement sleep, regardless of sex, ethnicity, age, cardiovascular medications, body mass index, and apnea/hypopnea index 23 and has been found to be associated with cardiovascular risk, including the risk of stroke 24.

### Chemotherapy and stroke

In our study, we found that patients with breast cancer who received chemotherapy had an elevated risk of stroke after propensity score matching, as expected. The associations between chemotherapy drugs and stroke have been studied.

Tamoxifen is associated with an increased risk of stroke, probably due to the higher risk of blood clot formation. A previously published meta-analysis showed an increased risk of all forms of stroke (relative risk [RR], 1.49; 95% CI: 1.16-1.90) 25.

Another study showed that patients with breast cancer who were treated with tamoxifen had an 82% increased risk of ischemic stroke and a 29% increased risk of any type of stroke, although the absolute risk was small 26. The authors suggested that further evaluation of prespecified cerebrovascular outcomes and clarification of the risk of stroke associated with tamoxifen use were needed.

The FDA added a warning to Avastin, which has been used to treat breast cancer, stating that it increases patients' risk of stroke. Research has shown that Avastin may cause severe strokes. It significantly increases the risk of cerebrovascular events, including ischemic stroke and hemorrhage stroke; the risk may vary with drug dosage and cancer type 27.

Another class of estrogen-targeting medications known as selective estrogen response modifiers (SERMs), which include toremifene and raloxifene, also affect the risk of stroke. Studies found that toremifene may be associated with a relatively lower risk of stroke. A retrospective analysis of adjuvant treatment trials involving toremifene and tamoxifen showed a significantly lower incidence of ischemic events with toremifene than tamoxifen 28.

A secondary end point analysis of an international, randomized, placebo-controlled clinical trial of postmenopausal women found that the risk of a fatal stroke was slightly higher in those assigned to receive raloxifene and those who already had risk factors for coronary artery disease 29. Breast cancer, chemotherapy and sleep disorders all increase the risk of stroke. Treatment decisions about medications should be made on the basis of the estimation of the absolute benefits and risks of different treatments with consideration of patient characteristics and multiple outcomes.

## Limitations

Because our study was performed using information from secondary databases, certain limitations and their statistical solutions should be mentioned. First, cancer is a major disease with high mortality rates. Given the possibility of the survival bias, the outcome of stroke needs to be observed after patients with breast cancer have survived treatment. Patients with a past history of stroke are at increased risk of stroke, and we excluded patients with a history of stroke. Furthermore, when performing an estimation of the risk of mortality due to stroke, cancer-related mortality had to be considered as a competing risk. Second, due to the limitations of the claims database, neurological deficits in stroke patients, brain images, laboratory data and poststroke sequelae could not be obtained. Third, some patients with sleep disorder did not seek medical treatment. Therefore, the patients with sleep disorder may be underestimated. However, based on large sample sizes presented in this study, our finding may not be affected.

## Conclusion

Cancer and ischemic stroke are common conditions. Breast cancer patients with sleep disorders have an increased risk of stroke. Treating sleep disorders and investigating the risk of stroke seems warranted in patients with breast cancer. The prompt diagnosis of stroke in breast cancer patients with sleep disorder is crucial for treatment and prevention.

## Figures and Tables

**Figure 2:**
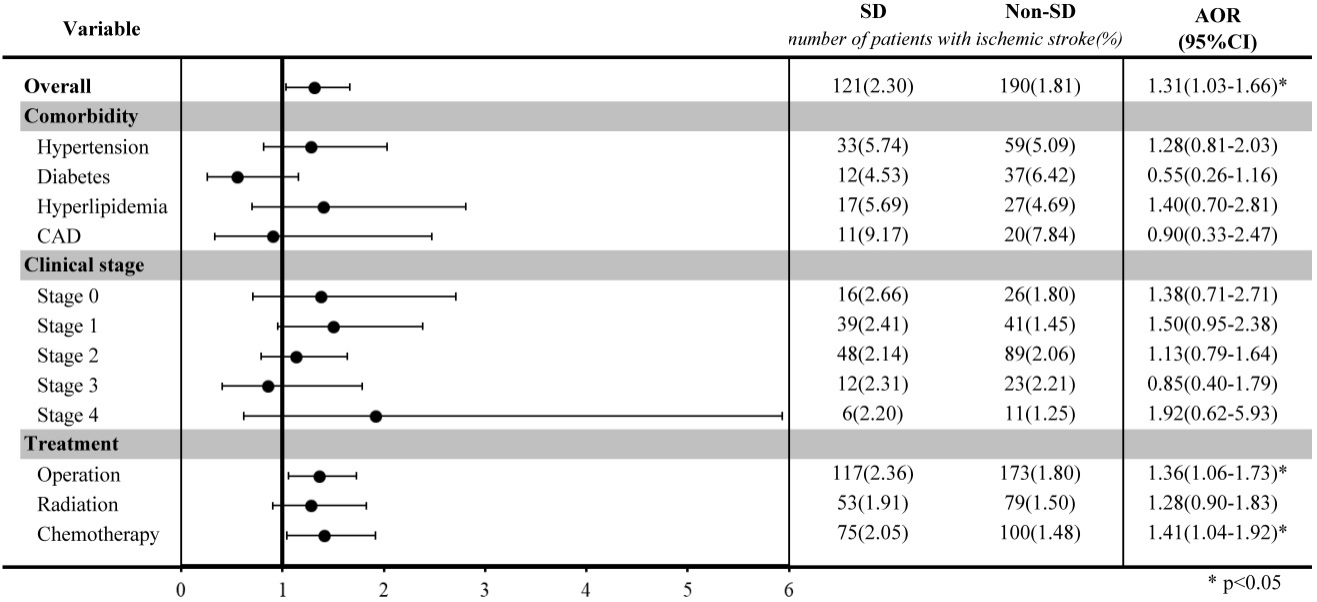
Stratified analysis of ischemic stroke risk in breast cancer patients with and without sleep disorders after matching by age and index year.

**Table 1:** Demographic characteristics and underlying comorbidities in the patients with and without sleep disorders

Variable	Before matching			After matching		
	SD (n =5295)	Non-SD (n =18036)	*p*-value	SD (n =5256)	Non-SD (n =10512)	*p*-value
**Age (years), mean±sd**	49.62±10.14	50.54±10.92	<.0001	49.55±10.14	49.50±10.29	0.8105
**Age (years), n (%)**						
<45	1649(31.14)	5216(28.92)	<.0001	1632(31.05)	3264(31.05)	1.0000
45-54	2142(40.45)	6950(38.53)		2124(40.41)	4248(40.41)	
55-64	1087(20.53)	4003(22.19)		1087(20.68)	2174(20.68)	
65-74	339(6.40)	1349(7.48)		335(6.37)	670(6.37)	
≥75	78(1.47)	518(2.87)		78(1.48)	156(1.48)	
**Comorbidity, n (%)**						
Hypertension	577(10.90)	2225(12.34)	0.0046	575(10.94)	1159(11.03)	0.8713
Diabetes	266(5.02)	1105(6.13)	0.0027	265(5.04)	576(5.48)	0.2490
Hyperlipidemia	302(5.72)	1084(6.01)	0.4065	299(5.69)	576(5.48)	0.5884
CAD	121(2.29)	457(2.53)	0.3061	120(2.28)	255(2.43)	0.5793
**Clinical stage, n (%)**						
0	607(11.46)	2600(14.42)	<.0001	601(11.43)	1443(13.73)	<.0001
1	1629(30.76)	4945(27.42)		1616(30.75)	2828(26.90)	
2	2267(42.81)	7142(39.60)		2247(42.75)	4321(41.11)	
3	519(9.80)	1788(9.91)		519(9.87)	1043(9.92)	
4	273(5.16)	1561(8.65)		273(5.19)	877(8.34)	
**Treatment, n (%)**						
Operation	4996(94.35)	16380(90.82)	<.0001	4959(94.35)	9605(91.37)	<.0001
Radiation	2795(52.79)	9223(51.14)	0.0348	2779(52.87)	5284(50.27)	0.0020
Chemotherapy	3689(69.67)	11208(62.14)	<.0001	3666(69.75)	6774(64.44)	<.0001
**Ischemic stroke, n (%)**	123(2.32)	302(1.67)	0.0019	121(2.30)	190(1.81)	0.0352
**Time to IS, mean±sd**	6.31±2.60	5.22±2.66	<.0001	6.29±2.59	6.00±2.76	<.0001
**Death, n (%)**	696(13.14)	2683(14.88)	0.0016	695(13.22)	1719(16.35)	<.0001
**Time to death, mean±sd**	6.38±2.58	5.26±2.66	<.0001	6.36±2.58	6.06±2.75	<.0001
**Time to SD, mean±sd**	2.01±2.11	--		2.00±2.10	--	

SD: sleep disorder; IS: ischemic stroke; sd: standard deviation.

**Table 2:** The risk of ischemic stroke in breast cancer patients with and without sleep disorders

Variable	Before match*				After match**			
	OR (95% CI)	*p*-value	AOR (95% CI)	*p*-value	OR (95% CI)	*p*-value	AOR (95% CI)	*p*-value
**Overall**								
Non-SD	Ref.		Ref.		Ref.		Ref.	
SD	1.40(1.13-1.73)	0.0020	1.58(1.27-1.96)	<.0001	1.29(1.02-1.63)	0.0333	1.31(1.03-1.66)	0.0260
**Comorbidity**								
Hypertension	3.78(3.08-4.64)	<.0001	1.53(1.18-1.97)	0.0013	1.67(1.26-2.20)	0.0004	1.37(1.00-1.88)	0.0489
Diabetes	3.39(2.62-4.40)	<.0001	1.31(0.97-1.76)	0.0765	1.62(1.16-2.27)	0.0049	1.31(0.91-1.88)	0.1511
Hyperlipidemia	2.84(2.16-3.73)	<.0001	1.06(0.78-1.46)	0.7046	1.55(1.10-2.18)	0.0133	1.18(0.80-1.73)	0.4034
CAD	4.45(3.19-6.21)	<.0001	1.65(1.15-2.37)	0.0069	2.13(1.40-3.23)	0.0004	1.78(1.15-2.74)	0.0095
**Clinical stage**								
0	Ref.		Ref.		Ref.		Ref.	
1	0.90(0.65-1.24)	0.5125	0.83(0.59-1.18)	0.2958	0.80(0.54-1.17)	0.2395	0.86(0.57-1.28)	0.4510
2	1.08(0.80-1.46)	0.5590	0.95(0.67-1.34)	0.7664	0.83(0.58-1.18)	0.2969	0.97(0.65-1.45)	0.8812
3	1.33(0.91-1.93)	0.1422	1.09(0.71-1.68)	0.6923	0.77(0.48-1.22)	0.2629	1.01(0.60-1.70)	0.9754
4	0.86(0.54-1.35)	0.5068	0.71(0.40-1.26)	0.2449	0.50(0.28-0.89)	0.0190	0.72(0.36-1.44)	0.3577
**Treatment**								
Operation	1.02(0.72-1.45)	0.9145	1.17(0.76-1.81)	0.4774	1.42(0.90-2.24)	0.1334	1.24(0.72-2.12)	0.4461
Radiation	0.68(0.56-0.83)	0.0001	0.88(0.72-1.08)	0.2237	0.95(0.75-1.20)	0.6629	0.99(0.78-1.26)	0.9328
Chemotherapy	0.72(0.60-0.88)	0.0010	0.94(0.74-1.19)	0.5926	0.76(0.60-0.96)	0.0232	0.75(0.57-0.99)	0.0450

OR: odds ratio; AOR: adjusted odds ratio; CI: confidence interval; *The OR was estimated using logistic regression analysis, and the AOR was adjusted by age, comorbidities, clinical stage, and treatment type. ** The OR was calculated using conditional logistic regression analysis after matching by age and index year, and the AOR was adjusted by comorbidities, clinical stage, and treatment type.
